# Objective Classification of mTBI Using Machine Learning on a Combination of Frontopolar Electroencephalography Measurements and Self-reported Symptoms

**DOI:** 10.1186/s40798-019-0187-y

**Published:** 2019-04-18

**Authors:** M. Windy McNerney, Thomas Hobday, Betsy Cole, Rick Ganong, Nina Winans, Dennis Matthews, Jim Hood, Stephen Lane

**Affiliations:** 1 Tahoe Institute for Rural Health Research, 10121 Pine Ave, PO Box 759, Truckee, CA 96160 USA; 2 Tahoe Forest Hospital, Truckee, CA USA; 3 Department of Neurological Surgery, University of California, Davis, Sacramento, CA USA

**Keywords:** EEG, mTBI, Machine learning

## Abstract

**Background:**

The reliable diagnosis of a mild traumatic brain injury (mTBI) is a pervasive problem in sports and in the military. The frequency and severity of each occurrence, while difficult to quantify, may impact long term cognitive function and quality of life. Despite the new revelations concerning brain disfunction from head injuries, individuals still feel pressure to remain on the field despite a debilitating injury. In this study, we evaluated the accuracy of a system that could be employed on the sidelines or in the locker room to provide an immediate objective mTBI assessment.

**Methods:**

Participants consisted of 38 individuals with a recent mTBI and 47 controls with no history of mTBI within the last 5 years. Participants were administered a simple symptom questionnaire, behavioral tests, and resting state EEG was measured using three frontopolar electrodes. An advanced machine learning algorithm called boosting was utilized to classify subjects into either injured or controls using power spectral densities on 1-min of resting EEG and the symptom questionnaire.

**Results:**

Results based on leave-one-out cross-validation revealed that the addition of EEG measurements boosted the accuracy to approximately 91 ± 2% compared to 82 ± 4% from the symptom questionnaire alone.

**Conclusion:**

This study demonstrated the potential benefit of including EEG measurements to diagnose suspected brain injury patients. This is a step toward accurate and objective classification measurements that can be implemented on the field as a future injury assessment tool.

## Key Points

- The combination of electroencephalography (EEG) and symptom questionnaires more accurately classified mTBI than questionnaires alone
- EEG can be used to reduce the subjectivity of the remove-from-play decision
- The boosting method in machine learning is a powerful tool for enhancing classification accuracy

## Background

Mild traumatic brain injury (mTBI) is highly prevalent, with an estimated 1.6–3.8 million sports-related concussions annually [1, 2] and as many as 320,000 concussions affecting military troops [3, 4]. According to the Centers for Disease Control (CDC), it is estimated that in the USA, mTBI costs are $17 billion annually, creating a huge burden on society [5]. The many possible symptoms of mTBI are, by themselves, fairly nonspecific and include headaches, dizziness, nausea, light/sound sensitivity, loss of consciousness, amnesia, irritability, cognitive changes, sleep disturbance, and emotional dysregulation. These symptoms can vary in severity and prevalence from person to person, making reliable diagnosis difficult. Despite new knowledge about the potential deleterious long-term effects of mild brain injury, athletes, their coaches, and military personnel experience added pressure to minimize symptom reporting, as to not let down teammates, or appear to be underperforming. Returning to the field with a brain injury is very dangerous as it could worsen the injury and lead to long-term changes in brain function. Unfortunately, relative to other common disorders of this magnitude, mTBI studies of optimal treatment and diagnosis are underfunded [6]. It is therefore extremely important to develop a simple, field-portable diagnostic tool that can objectively test for the presence of an mTBI.

Following a mTBI, people experience acute short-term symptoms (within a month) with a majority of the symptoms resolving in 2–3 weeks. Chronic post-concussive syndrome, which is the persistence of symptomology several months after injury, can have symptoms that are very similar to the initial acute phase, such as headache, memory loss, irritability, sensitivity to light, loss of concentration, and fatigue [7]. Approximately 15% of mTBI-diagnosed patients experience these persistent disabling difficulties [8]. It is challenging to determine why or how some patients end up with post-concussion syndrome, but it appears that gender and neurobehavioral test scores at the time of injury may be predictive of future chronic symptomology [9]. Importantly, research has shown that the presence of microstructure white matter lesions that failed to sufficiently heal at the time of another injury may be critical for the development of chronic post-concussive syndrome [10].

It is imperative that the brain is allowed adequate time to recover before another injury occurs. Autopsies on football players who likely received multiple successive concussions revealed an excess of amyloid-beta plaques and tau tangles [11]. Numerous blows to the head seem to be associated with clinical abnormalities and possibly chronic traumatic encephalopathy (CTE) [12]. Other possible long-term effects of successive concussions include other forms of dementia, mental health issues, and depression [13]. Brain recovery can extend beyond the clinical recovery time, so an improved neurological function index is needed [14]. Therefore, the consequences of sustaining multiple concussions, especially without adequate healing, could have serious long-term consequences, and researchers must determine a way to rapidly and adequately diagnose mTBI to prevent future progression of these diseases.

The post-concussion assessment and cognitive tests: Immediate Post-Concussion Assessment and Cognitive Testing (ImPACT; ImPACT Applications, San Diego, CA), Sports Concussion Assessment Tool, 5th Edition (SCAT5) [15], and the King-Devick [16] tests are the available as injury assessment tools. As part of these tests, a patient interview is also usually carried out, with questions about symptomology. Although these measures may be very useful, they can be influenced by trainer or player bias, so a better protocol should be adopted. Currently, patients are removed from the field or returned-to-play based on imprecise self-reported symptoms, without the measurement of the underlying pathophysiology. For example, patients can meet return-to-play criteria but still show abnormal brain measurements [17] or show changes in EEG measurements without any differences in the ImPACT, SCAT5, or King-Devick tests [18]. Because of these reasons, it is essential that the diagnostic test be as objective as possible and be difficult to bias the outcome. This is our motivation for developing an EEG concussion assessment tool.

The portable electroencephalograph has huge potential for a sideline mTBI diagnostic tool. Researchers are investigating the use of EEG and evoked potentials but have yet to adequately develop a diagnostic tool that has gained widespread use. Evoked potentials and EEG are a powerful assessment of brain activity and have the potential for mTBI diagnosis [19, 20], but this is difficult to set-up and requires a very long set of cognitive tasks and intense data post-processing. Diagnostic tools that require a large amount of time and subject someone with a brain injury to a lengthy set of cognitive tasks can be trying, and difficult for a patient that needs rest. On the other hand, a quantitative EEG (qEEG) approach could efficiently assess for a concussion in much less time. According to Haneef et al. [21], the acute, subacute, and chronic mTBI stages can be mapped out using qEEG measurements, suggesting that this technique could be used for diagnosis and monitoring recovery. Although analysis for unique EEG components in a mTBI is showing a lot of potential, it still requires the use of a large number of electrodes [22], making rapid measurements problematic.

We have utilized an inexpensive, portable, easily implementable EEG device that, with only three electrodes, can aid in acute mTBI diagnosis on the field, with only a few minutes of recording. Our advanced algorithm has enabled the differentiation between mTBI and no mTBI without the need for a baseline (pre-mTBI) measurement. Baseline measurements of every athlete or soldier are not always practical, so a diagnostic tool that does not require prior knowledge of personalized EEG activity is needed. Currently, in order to achieve meaningfully high prediction accuracies (> 90%), we are combining the EEG with patient-provided answers to a few questions about their symptoms. This publication describes our progress toward achieving high classification accuracy for individuals who recently sustained an mTBI vs those who have not, using simple EEG measurements while minimizing the number of non-EEG variables. This is a step toward a simple on-field EEG-only diagnostic test that reflects brain physiology and minimizes the need for questionnaires.

## Method

### Participants

Volunteers were recruited from local hospitals and sports fields. Typically, the participants came from high school and college sports clubs or recreational skiing. The remaining subjects received injuries from falls and from accidents. Our program has collected measurements on 189 participants consisting of both concussion and control participants, with a wide age range (18–80). For the purpose of this study, we chose to focus on the 18–32 year age range and use only participants who had measurements within 72 h of injury. We chose this age range to capture a representation of ages likely to be associated with sport and occupation-related injuries [23] and to reduce the variability in the sample that could be attributed to brain development (under 18) or neurodegeneration (older adult subject). Future studies will focus on adolescence, middle age, older adulthood, and potentially all age ranges. The control participants had no head injuries in the past 5 years, no history of moderate or severe head injuries, and no symptoms of chronic post-concussive syndrome. One control subject was removed because he/she reported five injury symptoms and one mTBI subject was removed because he/she reported no symptoms, both inconsistent with their assigned classes. This left us with a total of 85 participants, consisting of 27 females, 58 males, 38 mTBI (mean age = 22.79 ± 4.23), and 47 controls (20.90± 2.90).

All protocols and procedures were approved by an Institutional Review Board (IRB) under the guidelines of Aspire IRB (Santee, CA) and conformed to the ethical standards outlined in the Declaration of Helsinki. Each participant provided informed consent to participate in the study.

### Screening

Prior to participation, background information such as age, medical history, and prior history of concussions was documented. Participants were excluded from the study if they had a history of eye disease, strabismus, amblyopia, or another neurologic condition other than concussion. The subjects were capable of sitting motionless in a chair for 10 min, were able to follow simple directions, and were not in pain or otherwise impaired to the point that would interfere with the test. All of the subjects needed to have a Glasgow-Coma score of 15. Participants with a head injury were evaluated by trained medical professionals for a mTBI and subsequently referred to our research team. Control participants were screened for a history of TBI and any chronic symptoms from past mTBI as specified above.

### Materials

EEG measurements were all obtained using the wireless three-electrode B-Alert SleepProfiler system (Advanced Brain Monitoring, Carlsbad, CA) controlled by a tablet computer using software provided by Advanced Brain Monitoring. The three electrodes were located on the subject’s forehead in positions AF7, FpZ, and AF8 with a sampling rate at 256 Hz. The impedance was kept to below 40 kΩ. The beginning and end of individual tests are denoted by keyboard-entered event markers. The voltage vs. sample number and time from each electrode pair were stored on the computer for further analysis. The hardware has FDA 510(k) safety approval for a variety of applications.

### Procedure

Measurements were made in the following settings: hospital emergency room or a private examination room at a hospital, clinic, school, or ski-resort. In a few cases, the measurements were made at the participant’s home. In all cases, the noise level and other distractions were kept at a minimum level as allowed by the circumstances.

This study is a preliminary retrospective study as the measurements were made before the current analysis protocol was determined. The types of tests and testing order were slightly modified over the course of the study. A typical EEG test session consisted of a sequence such as (1) 1-min eyes open/resting state, (2) 1-min eyes closed/resting state, (3) King-Devick tests, (4) repeat 1, (5) repeat 2, (6) balance test, and (7) computer-based attention test. Keeping in mind that the eventual goal of the project is to develop an EEG-only test, we used just the 1-min resting state eyes closed tests, which has recently shown to have significant head injury diagnostic value [24]. All of the other tests were found to have low predictive power or motion-induced EEG artifacts and were thus eliminated from the analysis.

In addition to the above measurements, we used seven yes-no symptom questions. These questions were in reference to loss of consciousness, headache, nausea or vomiting, sensitivity to light, sensitivity to sound, confusion, and memory disfunction. These questions are similar to part of the ImPACT and SCAT5 tests. The subjects were also asked to rate the symptom severity on a scale of 0 to 6, 0 signifying no symptoms. The yes-no answers were used as variables in the analysis, and the eighth variable was the average numerical severity rating from all the symptoms.

## Analysis

### EEG Artifact Removal and Feature Extraction

The EEG data was bandpass filtered 0.1 Hz (high pass) and 100 Hz (low pass). Artifact removal included muscle movement (EMG), eye blinks, excursions, saturations, and spikes. Regions with excursions and saturated signals were marked and eliminated from the analysis. EMG artifacts were flagged by monitoring high-frequency EEG power in the 70–128 Hz band and low-frequency EMG in the 35–40 Hz band. A small number of blink-like signal shapes that were detected using the algorithm by Chang, Cha, Kim, and Im [25] and removed. All detected artifacts were manually inspected and adjustments were made to the eliminated data region if appropriate.

Matlab software version R2017a (The Mathworks, Natick) was used to perform feature extraction and data analysis for the AF7-FpZ and AF8-FpZ voltages. Power spectral densities (PSD) were calculated from 1 to 40 Hz in 1-Hz frequency and 1-s time bins. The conventional EEG bands (delta 1–3 Hz, theta 3–7 Hz, alpha 8–13 Hz, beta 13–30 Hz, sigma 12–15 Hz, and gamma 25–40 Hz) were obtained by summing the 1-Hz frequency bins. As a method of data smoothing, for each 256 samples (1-s time bin), the PSD was obtained from the average of three overlapping 1-s time windows, the 1-s bin, and the two time bins surrounding it. The observational variables used were the base-10 logarithm of the power spectral density (PSD) in each of the standard EEG bands averaged over the full testing period.

### Algorithm

The Matlab function *fitensemble* was used to create the classification model. The binary classification (mTBI or healthy control) was performed using the supervised learning method *TotalBoost* [26, 27]. This ensemble statistical learning uses a large collection of classifiers to boost the accuracy over that obtained by a single classifier [28]. For this algorithm, the binary class (mTBI vs control) for each subject is provided, and the goal is to sufficiently train the algorithm so that it can accurately classify new subject data into mTBI or control. Boosting is a meta-algorithm that works with a collection of other classifiers, such as decision trees. With each iteration, boosting brings in a new decision tree to improve areas of weak performance within the algorithm. Typically, several hundred iterations are employed before the algorithm converges. In our case, we used 200 decision trees.

Up to 20 observational variables were used in the classification analysis. These variables included the delta, theta, alpha, beta, sigma, and gamma bands from the A7-FpZ and A8-FpZ voltages (totaling 12 EEG variables). The yes/no answers to the symptom questions and the average of the numerical intensities (on a 0–6 scale) of the seven symptoms were also included. Thus, the number of variables was between 12 (no non-EEG variables) to 20 (12 EEG variables + 8 non-EEG variables).

Leave-one-out cross-validation was used to estimate the accuracy of the classification. Here, for our set of 85 observations, 84 observations were used to train the algorithm. The left-out test observation was then predicted by the trained algorithm. This procedure was repeated for each of the observations. Although it is more common to cross-validate with two sets of participants (training set and testing set), we were restricted by our sample size and turned to the leave-one-out approach, which is accepted as a method for testing algorithms [29]. A future study with a larger sample size to allow for the 1/3 testing 2/3 training subject partitioning is a possible next step. The resubstitution, or training, accuracy is determined when the full set of observations is used for training as well as for testing [30]. In all cases, our resubstitution accuracy was found to be 100%. For a modern powerful machine learning algorithm and a small data set such as ours, this not an unexpected result. For much larger data sets, with overlapping distributions in variable space, this would have been a signature of overfitting. The leave-one-out cross validation more accuracy represents model performance and, for this study, was the proportion of correctly predicted observations. With this approach, one possible sign of overfitting of the training algorithm is the combination of high resubstitution accuracy but low cross-validation accuracy. As discussed below, this was not the case for our study.

## Results

**Fig. 1:**
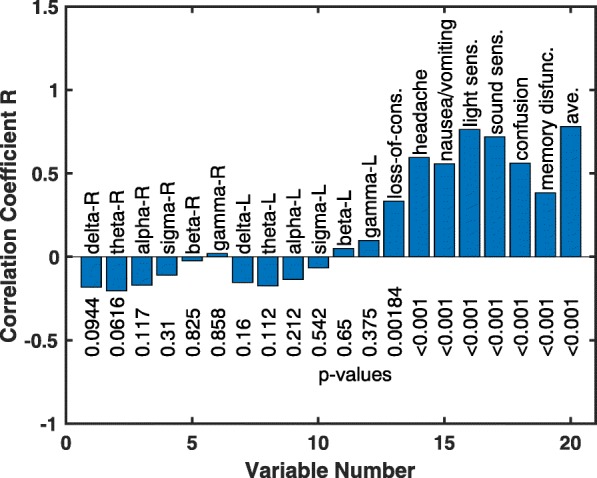
The correlation R coefficients and corresponding *p* values are obtained by comparing each observational variable (data matrix column) with the class vector. The EEG variables are labeled by the conventional frequency band names with R and L indicating the right and left electrodes

In selecting the symptom variables to use in the classification algorithm, the correlation coefficient *R* and corresponding *p* value comparing each variable column in the data matrix with the class vector. Figure 1 displays the results. It is seen that most EEG variables have weak anti-correlations with the class matrix while the symptom variables have relatively strong correlations.

As a second test of predictive value, all 256 possible combinations of symptom variables combined with the full set of EEG variables were investigated using the classification algorithm to determine what variable set gave the most accurate prediction. It was found that the average severity variable with any combination of the confusion, light sensitivity, and sound sensitivity variables gave the best results when the number of symptoms was limited to three or four. When the number of symptoms was limited to five or six symptoms, the most predictive variables also included loss of consciousness and headache.

To demonstrate that EEG variables can boost predictive accuracy for a small number of symptoms, the three-member symptom set—confusion, light sensitivity, and average severity—were used to illustrate this point. The accuracy for the combined EEG and three-symptom set was found to be near 91%.

**Fig. 2:**
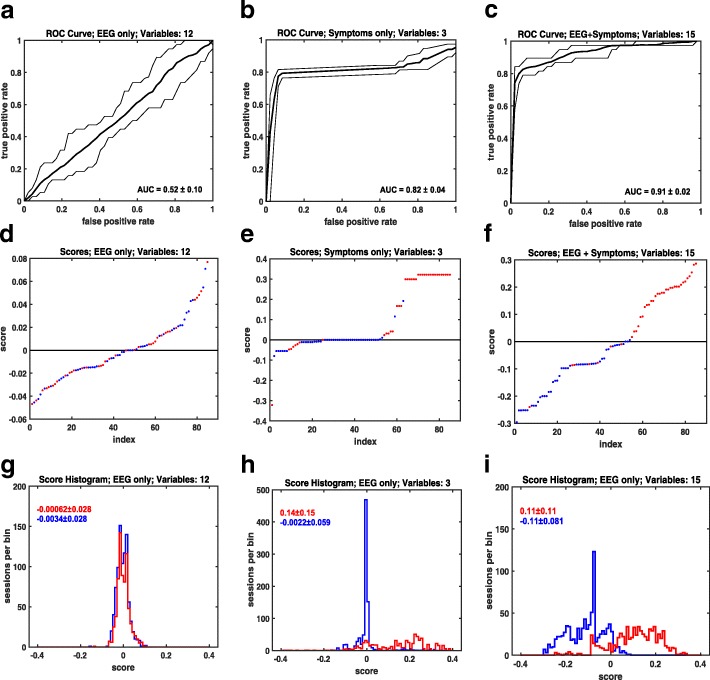
ROC analysis results for the TotalBoost classification algorithm applied to the set of 85 subjects made of injured and control classes. Three cases are shown: EEG-only (**a**, **d**, **g**), symptoms-only (**b**, **e**, **h**), and EEG plus symptoms (**c**, **d**, **i**). The first row plots the ROC curves, the second row plots the ordered scores, and the third row shows the score distribution for the three cases. The AUC average and maximum/minimum variation are given in the ROC plots (top). The distribution means and standard deviations are shown in the distribution plots (bottom)

Although the classification accuracy is often used to gauge the performance of a classification algorithm, it is argued in the literature [31–33] that receiver operator characteristic (ROC) curve analysis gives a more complete representation of the algorithm effectiveness. Three cases are considered in Fig. 2: (1) the 12 EEG variables (A, D, G), (2) the three symptom variables—confusion, light sensitivity, severity average (B, E, H), and both the EEG and the symptom variables (C, F, I). The top row shows the ROC curves for the three cases. The area under the curve (AUC) is used as a metric for comparison. In our analysis, the predictions and scores are obtained from the test subjects (subjects that have been left out in the cross-validation). Scores that have values greater than the class boundary at zero indicate mTBI predictions while those less than zero refer to control predictions. Scores with larger absolute values indicate higher posterior probabilities.

For each set of prior probabilities, a different set of predictions and scores are produced, leading to slightly different ROC curves. Since the prior probabilities are unknown, each of these curves is equally valid. We generated a family of 19 ROC curves for each case by varying the relative class weights by integer values from 1 to 10. We then took the average of these 19 curves as shown in graphs A, B, and C. The maximum and minimum values are also plotted to show the range of variability. The average AUC values are approximately 0.52, 0.82, and 0.91 for the EEG-only, symptom-only, and EEG plus symptom cases, respectively.

In the second row, we plot the ordered scores for each of the three cases. Only the instance of equally weighted classes is shown but the other unequal weighting instances are similar in shape. The injured subjects (positive) are shown in red, healthy (negative) in blue. It is noted that the score values for the symptom-only case (E), while giving relatively accurate predictions, have a large fraction of small score values, indicating a weak level of predictive robustness. On the other hand, the combined variable case (F) does not exhibit this feature, having the majority of the score values far from the class boundary.

The third row displays plots of the score distributions. As before red indicates the distribution of injured subject scores and blue corresponds to controls. The EEG-only plot (G) shows nearly overlaying distributions centered at the class boundary. The symptom-only case (H) shows a distribution means separated by 0.14 score units. These distributions are in contrast to the EEG + symptom which exhibits a greater class separation of 0.22 units.

This result exhibits the nonintuitive but well-established effect where one set of observed variables alone has little or no predictive value, but when combined with a second set of variables, the correlations between the members of these sets result in stronger predictive power of the resultant latent variables compared to that of either set alone. We checked that the result was not due to noise or added ROC points by randomizing the EEG variables across subject measurements. As expected, the randomization removed the correlation of the EEG variables with symptom variables and resulted in the combined EEG+symptoms AUC value being the same as that of the symptoms alone.

## Discussion

Our machine learning algorithm method was able to correctly classify all of the participants during the training stage. The participants in the study included those who sustained a head injury and an mTBI diagnosis within 72 h of measurement, and controls, thus paving the way for a diagnostic tool. Leave-one-out validation resulted in classification accuracy of approximately 91 ± 2%, showing that our simple and portable EEG protocol is capable of assessing brain injury with high accuracy. In addition, the ROC analysis demonstrated the dangers (low confidence predictions) of relying on symptom questions alone. Our protocol consisted of a handful of symptom questions and only 1 min of EEG recordings on three frontal electrodes. The EEG device is wireless and controlled with a small tablet. Therefore, the test from the current study can easily be implemented on the sideline, locker room, or in a military deployment situation.

Currently, the concussion assessment protocol on the sports field consists of symptom questions and behavioral measurements. The symptom questions from our data provided 75–82% accuracy with weak predictive robustness resulting in marginal performance for practical applications. The addition of EEG data boosted the classification accuracy and ROC AUC to approximately 91%. Supplementing the question-based approach with EEG data can thus increase accuracy and help reduce any bias, resulting in a more appropriate remove-from-play judgment call. Importantly, research has shown that EEG measurements can pick up the effects of an mTBI when behavioral tests show no differences [14, 34]. We therefore suspect that future analysis algorithms will only increase the EEG contribution to the decision accuracy, thus avoiding a misdiagnosis based on subjective behavioral tests. This is an important step in reducing the long-term health consequences of individuals when he/she has suffered a brain injury.

The current study focused on those who sustained an injury within 72 h, which is far from a sideline measurement immediately following injury. The symptom profile of a concussion can change over time, with some symptoms not readily apparent until 24 h post injury [35]. Therefore, future studies should aim at shortening the time window post injury for a more accurate on-field diagnostic assessment tool. Future research could also compare EEG data from those immediately following injury (within 24 h) and a little farther out from injury (24–72 h) to better understand the difference between an immediate measurement and a brain in the beginning stages of healing.

Developing tools that facilitate the accurate diagnosis of a mTBI is of the upmost important as many individuals under-report symptoms [36] and believe it is fine to play through a concussion even with background knowledge of the potential dangers [37]. The cascade of events and metabolic crisis that occurs in the brain following a mTBI sets the brain up for a vulnerability to subsequent injury that could result in a chronic brain disease [38]. The period of vulnerability is unknown as it depends on many factors, such as severity, but it is clear that a subsequent injury occurring prior to full physiological recovery can be detrimental to the brain. It is therefore important to create a means of measuring the initial injury and the recovery of that injury. We suspect that the development of measurement techniques that can differentiate in mTBI injury severity is critical in the investigation of injury duration [14], but our study focuses on a yes-no diagnosis for the purposes of an accurate and effective remove-from-play protocol. In professional sports, more of an emphasis on this is underway, but in recreational and amateur sports leagues, there are not necessarily trained medical personnel (e.g., athletic trainers) present at all times [39]. A portable simple measurement EEG tool may help resolve this issue.

Other approaches have been able to classify concussions with high accuracy using an EEG-based technique [14, 40, 41]. However, these approaches have used higher density EEGs and longer testing times [40], which are more difficult to implement on a sideline. Other data collection paradigms have focused on the recommendation for brain scans as a follow-up test for a possible brain bleed, rather than classifying a concussion [20]. Our study provides the unique approach of combining simple EEG measurements paired with advanced quantitative analysis to provide a highly accurate injury assessment tool. There has been a debate in the literature regarding the validity of EEG, specifically qEEG in the diagnosis of an mTBI as EEG research has yet to confirm or rule out a mTBI [21]. Recent research has deployed a three-classification system: concussion, no concussion, and no decision due to low confidence [42], which provides a viable solution to the issue about EEG classification validity and will be utilized in our future studies. This method will likely be more robust to situations where individuals answer symptom questioners that are inconsistent with their potential injury or lack of injury as the case in this manuscript. Overall, this project is a step closer to achieving the goal of a highly accurate injury assessment tool.

Recommendations for research on EEG and mTBI include using advanced analytical techniques to help reduce limitations in the diagnostic capabilities of EEG and qEEG [43]. We have followed this recommendation and deployed a machine learning algorithm technique, resulting in a classification with high accuracy. Although the EEG data, in the present case, on its own will likely not provide confidence to effectively diagnose an individual, a simple EEG measurement paired with a symptom questionnaire can be an effective metric. Future research is expected to uncover the key to an effective, deployable EEG technique for highly accurate EEG-based mTBI classification that requires very few or even a complete absence of non-EEG variables.

## Conclusions

The current study employed simple EEG measurements in addition to a symptom questionnaire on participants who recently experienced a concussion and healthy volunteers. Machine learning was utilized to classify our participants based on symptoms alone or symptoms with EEG, and results revealed that the addition of EEG boosted accuracy to approximately 91 ± 2% from 82 ± 4% for the symptom questionnaire alone. Although the improvement is modest this demonstrates progress toward accurate and objective on-field diagnostics. The addition of a physiological brain measurement will help ensure that sports players and military personnel minimize the chance of long-term damage.

## Abbreviations

AUD: Area under the curve
CDC: Centers for Disease Control
CTE: Chronic traumatic encephalopathy
EEG: Electroencephalography
EMG: Electromyogram
ImPACT: Immediate Post-Concussion Assessment and Cognitive Testing
IRB: Institutional Review Board
mTBI: Mild traumatic brain injury
PSD: Power spectral density
qEEG: Quantitative electroencephalography
ROC: Receiver operator characteristic
SCAT5: Sports Concussion Assessment Tool, 5th Edition
